# In-feed resin acids reduce matrix metalloproteinase activity in the ileal mucosa of healthy broilers without inducing major effects on the gut microbiota

**DOI:** 10.1186/s13567-019-0633-3

**Published:** 2019-02-22

**Authors:** Marisol Aguirre, Juhani Vuorenmaa, Eija Valkonen, Hannele Kettunen, Chana Callens, Freddy Haesebrouck, Richard Ducatelle, Filip Van Immerseel, Evy Goossens

**Affiliations:** 1 Department of Pathology, Bacteriology and Avian Diseases, Ghent University, Salisburylaan 133, 9820 Merelbeke, Belgium; 2 Hankkija Ltd, Peltokuumolantie 4, 05800 Hyvinkää, Finland

## Abstract

The chicken gut is constantly exposed to harmful molecules and microorganisms which endanger the integrity of the intestinal wall. Strengthening intestinal mucosal integrity is a key target for feed additives that aim to promote intestinal health in broilers. Recently, dietary inclusion of resin-based products has been shown to increase broiler performance. However, the mode of action is still largely unexplored. Coniferous resin acids are known for their anti-microbial, anti-inflammatory and wound-healing properties, all properties that might support broiler intestinal health. In the current study, the effect of pure resin acids on broiler intestinal health was explored. Ross 308 broilers were fed a diet supplemented with coniferous resin acids for 22 days, after which the effect on both the intestinal microbiota as well as on the intestinal tissue morphology and activity of host collagenases was assessed. Dietary inclusion of resin acids did not alter the morphology of the healthy intestine and only minor effects on the intestinal microbiota were observed. However, resin acids-supplementation reduced both duodenal inflammatory T cell infiltration and small intestinal matrix metalloproteinase (MMP) activity towards collagen type I and type IV. Reduced breakdown of collagen type I and IV might indicate a protective effect of resin acids on intestinal barrier integrity by preservation of the basal membrane and the extracellular matrix. Further studies are needed to explore the protective effects of resin acids on broiler intestinal health under sub-optimal conditions and to elaborate our knowledge on the mechanisms behind the observed effects.

**Electronic supplementary material:**

The online version of this article (10.1186/s13567-019-0633-3) contains supplementary material, which is available to authorized users.

## Introduction

The chicken intestinal mucosa represents a barrier that protects the body against antigens, microbial toxins, invasive pathogens and toxic molecules taken up with the diet. Numerous studies show that disturbances in the intestinal ecosystem have profound consequences on animal performance, health and welfare [1–3].

Regulatory and consumer pressure to reduce antimicrobial usage in production animals has encouraged studies on alternatives to antimicrobials. Most of these alternatives are feed additives that aim to steer towards a healthy gut microbiota, and towards preserving intestinal integrity and thus reducing excessive inflammatory responses [4, 5]. Examples include short and medium chain fatty acids (e.g. butyric and caproic acid [6, 7]), dietary fibers [8–10], probiotics [11, 12], and even vaccines [13–15] and bacteriophages [16, 17]. In addition, plant-derived phytochemicals have shown promising performance-enhancing effects in broilers as well as antimicrobial activities against pathogenic bacterial species [18, 19].

Resins derived from coniferous trees are phytochemicals that were used since ancient times in Asian and Scandinavian traditional human medicine. These resin-based products are mainly used for treating wounds, sores, pressure ulcers and a variety of other skin problems [20–22]. Recent scientific research has confirmed the efficacy of these compounds, both in human clinical trials as well as in animal models and in vitro [20, 23–26]. The effects of resins are presumed to be powered by their characteristic cocktail of terpenes (including abietic, dehydroabietic, neoabietic, isopimaric, levopimaric and palustric acids), which display a wide range of pharmacological properties, including, amongst other, anti-microbial, anti-tumor and anti-inflammatory activities [20, 27–34].

In the last years, resin-based products have been explored as feed components to improve and maintain intestinal health of broilers [35–37]. While the integrity of the chicken gut is of key importance in broiler health and performance, challenges that affect epithelial integrity are continuously encountered (coccidia, bacterial pathogens such as *Clostridium perfringens*, feed antigens and mycotoxins, amongst others) [1, 38]. Loss of intestinal mucosal integrity leads to intestinal inflammation, as antigens will initiate a cascade of molecular events that lead to pro-inflammatory cytokine expression. The intestinal microbial composition is also crucial in, either or not, maintaining gut homeostasis. Given the anti-microbial and anti-inflammatory properties of resin, potential beneficial effects in the avian gut may be expected. Indeed, inclusion of a resin-based product in the diet of broiler chickens significantly increased broiler performance when birds were under *C. perfringens* challenge [35, 36]. Furthermore, even under unchallenged conditions broiler performance was increased by inclusion of resin-based products in a commercial diet supplemented with or without chemical coccidiostats [35, 37]. However, little is known about the mechanism by which these resin-based products influence broiler performance. The described benefits do not seem to be associated with the feed type [35]. Moreover, the potential effect on broiler microbiota remains unclear. Unfortunately, all broiler studies described so far used a resin-based product containing a combination of resin-specific tall oil fatty acids (~90%) and resin acids (~8%). As both the tall oil fatty acids fraction as well as the resin acids fraction of these resin-based products can have an effect on bird performance, it is still unclear whether one or the other fraction, or the combination, is essential to gain the observed results.

The purpose of this study was to evaluate the effect of dietary supplementation of pure resin acids on broiler intestinal health under non-challenged conditions. Therefore, we focused on the effect of resin acids on both the intestinal microbiota as well as the intestinal tissue morphology and collagenolytic activities, since host metalloproteinases involved in collagen breakdown are known to play a crucial role in maintaining intestinal mucosal structure.

## Materials and methods

### The composition of the resin acids mixture

A mixture of natural resin acids from Scotch pine (*Pinus sylvestris*) and Norway spruce (*Picea abies*) was obtained from Hankkija Oy (Hyvinkää, Finland) and produced by Forchem (Rauma, Finland). This pure resin acids product is a derivative of the commercial product Progres® from Hankkija Oy (patent no FI124918) which was used in previously published broiler studies and contains about 90% free fatty acids and 8%–9% resin acids [35–37]. The resin acids mixture used in this study represents the pure resin acid fraction which contains mainly abietic acid and dehydroabietic acid (Table 1).

**Table 1:** **Composition of the mixture of coniferous resin acids**

Major resin acids	w%
Abietic acid	47.30%
Dihydroabietic acid group	1.80%
Dehydroabietic acid	22.60%
Neoabietic acid	0.90%
Dehydrodehydroabietic acid	0.80%
Nordehydroabietc acid	–
7,9 (11)-abietic acid	5.30%
13-B-7,9 (11)-abietic acid	4.50%
8,12-abietic acid	1.90%
Secodehydroabietic 1	–
8,15-pimaric acid	1.40%
Pimaric acid	0.50%
Isopimaric acid	3.40%
Sandaracopimaric acid	1.40%
Levopimaric acid	–
8,15-isopimaradien-18-oic acid	–
8,15-Pimaradien-18-oic acid	n.a.
Palustric acid	6.80%
Abietatetranoic acid	n.a.

### Birds, housing and experimental treatment

The study was undertaken following the guidelines of the ethics committee of the Faculty of Veterinary Medicine, Ghent University, in accordance with the EU Directive 2010/63/EU. Twenty, 1-day-old Ross 308 broilers were obtained from a local hatchery and housed in two pens (10 chickens per pen) on wood shavings. The animals were not vaccinated. Water and commercial starter feed (day 1–10) or grower feed (day 11–22) were provided ad libitum (Table 2). The control group received the standard non-supplemented diet, whereas the birds in the treatment group were fed the same feed supplemented with 200 mg resin acids/kg feed throughout the whole trial period. This resin acids supplementation level is in the same order of magnitude as used previously in studies assessing the effect of resin-based products on broiler performance [35–37]. At day 22, all birds were euthanized for sampling. Ileal and caecal content were stored at −70 °C for microbiota composition analysis (16S rRNA sequencing) and short-chain fatty acid (SCFA) analysis. Intestinal tissue from the different segments of the small intestine was snap frozen in liquid nitrogen and stored at −20 °C until protein extraction was performed for MMP analysis. Additionally, duodenal and ileal tissue samples were collected and fixed in 4% phosphate buffered formaldehyde for histological analysis.

**Table 2:** **Composition and nutritional value of the diets**

	Starter diet 0–10 days^a^	Grower diet 11–22 days^b^
Nutrients (w%)		
Crude protein	19.0%	18.0%
Crude fat	5.5%	4.8%
Crude ash	5.5%	5.5%
Cellulose	3.5%	3.2%
Lysine	1.09%	0.97%
Methionine	0.50%	0.45%
Calcium	0.85%	0.85%
Phosphor	0.62%	0.53%
Sodium	0.15%	0.15%
Nutritional additives/kg		
E672 Vitamin A	12 500 IU	
E671 Vitamin D3	2500 IU	
3a700 Vitamin E (all-rac alpha tocopheryl acetate)	80 mg	
E1 Iron (Iron (II) sulphate monohydrate)	51 mg	
E2 Iodine (Calcium iodate, anhydrous)	2.11 mg	
E4 Copper (Copper (II) sulfate pentahydrate)	10 mg	
E5 Manganese (Manganese (II) oxide)	75 mg	
E6 Zinc (Zinc oxide)	70 mg	
E8 Selenium (Sodium Selenite)	0.30 mg	
Zootechnical additives/kg		
4a1640 6-phytase (EC 3.1.3.26)	250 FTU	
Technological additives/kg		
E324 Ethoxyquin	33 mg	
E321 BHT	66 mg	
Coccidiostats and histomonostats/kg		
E763 Lasalocid-sodium	125 mg	

^a^Composition containing wheat, soy, corn, rice bran, wheat gluten, soy oil, corn gluten, palm oil, calcium carbonate, monocalcium phosphate, corn bran, sodium chloride, sodium bicarbonate.

^b^Composition containing wheat, soy, corn, wheat gluten, sunflower seeds, soy oil, palm oil, calcium carbonate and monocalcium phosphate.

### DNA extraction

DNA was extracted from intestinal content using the CTAB method as previously described by Griffiths et al. and De Maesschalck et al. with minor modifications [8, 39]. In brief, 100 mg of caecal content or 200 mg of ileal content was suspended in 0.5 mL CTAB buffer (hexadecyltrimethylammonium bromide > 98% (Sigma Aldrich, St. Louis, MO, USA) 5% (w/v), 0.35 M NaCl, 120 nM K_2_HPO_4_) and 0.5 mL phenol–chloroform-isoamyl alcohol (25:24:1). The mixture was homogenized by grinding (2×) with 0.5 g unwashed glass beads (Sigma-Aldrich) in a bead beater (1.5 min, 22.5 Hz; TissueLyser; Qiagen, Hilden, Germany) with a 30 s interval between shakings. Samples were centrifuged for 10 min at 8000 rpm and 300 µL of the supernatant was transferred to a new tube. A re-extraction from the remaining content was performed by adding 0.25 mL CTAB buffer and homogenizing and centrifuging the sample as described above. An equal volume (600 µL) of chloroform-isoamyl alcohol (24:1) was added to the supernatant collected in order to remove the phenol from the samples. The mixture was further centrifuged at 16 000 *g* for 10 s. Nucleic acids were precipitated with 2 times the volume of polyethyleenglycol-6000 solution (30% w/v; 1.6 M NaCl) for 2 h at room temperature. Samples were centrifuged (13 000 *g*, 20 min) and washed with 1 mL ice-cold ethanol (70% v/v). The pellet obtained was further centrifuged (13 000 *g*, 20 min), dried and resuspended in 100 µL de-ionized water (LiChrosolv Water, Merck, Darmstadt, Germany). The quality and the concentration of the DNA was examined spectrophotometrically (NanoDrop, Thermo Scientific, Waltham, MA, USA).

### 16S rRNA gene amplicon sequencing

To characterize the taxonomic groups in the caecal and ileal microbiota of the chickens, the V3-V4 hypervariable region of 16s rRNA gene was amplified using the gene-specific primers S-D-Bact-0341-b-S-17 (5′-TCGTCGGCAGCGTCAGATGTGTATAAGAGACAGCCTACGGGNGGCWGCAG-3′) and S-D-Bact-0785-a-A-21 (5′-GTCTCGTGGGCTCGGAGATGTGTATAAGAGACAGGACTACHVGGGTATCTAATCC-3′) [40]. Each 25 µL PCR reaction contained 2.5 µL DNA (~5 ng/µL), 0.2 µM of each of the primers and 12.5 µL 2 × KAPA HiFi HotStart ReadyMix (Kapa Biosystems, Wilmington, MA, USA). The PCR amplification consisted of initial denaturation at 95 °C for 3 min, followed by 25 cycles of 95 °C for 30 s, 55 °C for 30 s, 72 °C for 30 s and a final extension at 72 °C for 5 min. The PCR products were purified using CleanNGS beads (CleanNA, Waddinxveen, The Netherlands). The DNA quantity and quality was analyzed spectrophotometrically (NanoDrop) and by agarose gel electrophoresis. A second PCR step was used to attach dual indices and Illumina sequencing adapters in a 50 µL reaction volume containing 5 µL of purified PCR product, 2× KAPA HiFi HotStart ReadyMix (25 µL) and 0.5 µM primers. The PCR conditions were the same as the first PCR with the number of cycles reduced to 8. The final PCR products were purified and the concentration was determined using the Quantus double-stranded DNA assay (Promega, Madison, WI, USA). The final barcoded libraries were combined to an equimolar 5 nM pool and sequenced with 30% PhiX spike-in using the Illumina MiSeq v3 technology (2 × 300 bp, paired-end) at the Oklahoma Medical Research center (Oklahoma City, OK, USA).

### Bioinformatics and statistical analysis of 16S rRNA gene amplicon data

Demultiplexing of the amplicon dataset and deletion of the barcodes was done by the sequencing provider. Quality of the raw sequence data was checked with the FastQC quality-control tool (Babraham Bioinformatics, Cambridge, United Kingdom) followed by initial quality filtering using Trimmomatic v0.38 by cutting reads with an average quality per base below 15 using a 4-base sliding window and discarding reads with a minimum length of 200 bp [41]. The paired-end sequences were assembled and primers were removed using PANDAseq [42], with a quality threshold of 0.9 and length cut-off values for the merged sequences between 390 and 430 bp. Chimeric sequences were removed using UCHIME [43]. Open-reference operational taxonomic unit (OTU) picking was performed at 97% sequence similarity using USEARCH (v6.1) and converted to an OTU table [44]. OTU taxonomy was assigned against the Silva database (v128, clustered at 97% identity) [45] using the PyNast algorithm with QIIME (v1.9.1) default parameters [46]. OTUs with a total abundance below 0.01% of the total sequences were discarded [47], resulting in an average of approximately 48 336 reads per sample. Alpha rarefaction curves were generated using the QIIME “alpha_rarefaction.py” script and a subsampling depth of 10 000 reads was selected. One ileal and 1 caecal sample from the resin acids-supplemented group were excluded from further analysis due to insufficient sequencing depth.

Further analysis of alpha diversity (Observed OTUs, Chao1 richness estimator and Shannon diversity estimator) and beta diversity (Bray–Curtis dissimilarities) were performed using the *phyloseq* [48] pipeline in R (v3.4.3). Normality of the alpha diversity data was tested using the Shapiro–Wilk test. A t-test was used for normal distributed data, whereas the Mann–Whitney U test was used for not normal distributed data. Differences in beta diversity were examined using the *anosim* function from the *vegan* package. Differences in relative abundance at the phylum level were assessed using the two-sided Welch *t*-test from the mt wrapper in *phyloseq*, with the p-value adjusted for multiple hypothesis testing using the Benjamini–Hochberg method. To detect differentially abundant taxa between the different diet groups, DESeq 2 was applied on the non-rarified community composition data for either caecal or ileal communities [49]. Significant differences were obtained using a Wald test followed by a Benjamini–Hochberg multiple hypothesis correction. For all tests, a *p*-value < 0.05 was considered significant.

### Metabolic function prediction of the microbial communities

To gain more insight into the effect of resin acids on the possible functional pathways of the microbial communities, the functional composition was predicted using PICRUSt (Phylogenetic Investigation of Communities by Reconstruction of Unobserved States; [50]). PICRUSt uses precomputed ancestral state reconstructions based on the Greengenes database. Therefore, OTU picking was reperformed as described above with following modifications: closed-reference OTU picking was used, and OTU taxonomy was assigned against the Greengenes database (v 13.5) [51] after which the OTU counts were normalized by their expected 16 s copy number using QIIME [52, 53]. Metagenome predictions were performed against the KEGG database (Kyoto Encyclopedia of Genes and Genomes; [54]), after which the resulting KEGG orthologues were further summarized as functional pathways using PICRUSt.

### SCFA (acetate, propionate, butyrate, valerate) quantification

The amount of acetate, propionate, butyrate and valerate were quantified using gas chromatography as previously described [55]. In short, SCFA were extracted from caecal content using diethyl ether. 2-Methyl hexanoic acid (99%) was added to each sample as internal standard. The extracts were analyzed using a GC-2014 gas chromatograph (Shimadzu, ‘s-Hertogenbosch, the Netherlands), equipped with a capillary fatty-acid free EC-1000 Econo-Cap column (Alltech, Laarne, Belgium).

### Intestinal morphology

Intestinal morphology was analyzed by measuring villus length and crypt depth. Formalin-fixed intestinal tissue samples were embedded in paraffin and sectioned at 5 µm. To analyze the intestinal morphology, duodenal tissue was stained with hematoxylin and eosin. Villus height and crypt depth were assessed using a PC-based image analysis system (Leica Application Suite V4, LAS V4.; Leica, Diegem Belgium). Villus height was measured from the tip of the villus to the crypt-villus junction. Crypt depth was measured from its base up to the crypt-villus invagination. Measurements were performed in duodenal sections by random measurement of 10 villi/crypts per section, after which the average per animal was calculated.

### CD3 immunohistochemistry

Slides for immunohistochemical staining for CD3^+^ cells were automatically deparaffinized (Shandon Varistain-Gemini) before antigen retrieval with a pressure cooker in citrate buffer (10 mM, pH 6). Endogenous peroxidase activity was blocked by treating the slides with peroxidase blocking reagent (S2023, Dako, Glostrup, Denmark) for 5 min. The presence of T-cells (CD3-positive cell abundance) in intestinal tissue from both duodenum and ileum was evaluated using polyclonal primary antibodies against CD3 (A0452, Dako, 1:100 dilution, 30 min at room temperature), followed by incubation with a secondary labelled polymer-HRP anti-rabbit (Envision + System-HRP (DAB) (K4011), 30 min at room temperature). Slides were evaluated using the computer based image analysis program, LAS V4.1. The CD3^+^ area percentage in either the duodenal or ileal tissue was quantified using three representative fields of view per intestinal section.

### Intestinal tissue lysates

Proteins were extracted from the small intestinal tissue (duodenum, jejunum and ileum) using mechanical lysis. In brief, intestinal tissues (~30 mg) were homogenized in 400 µL TBS-1% NP-40 (50 mM Tris/HCl, pH 8.0, 150 mM NaCl and 1% (v/v) NP-40, supplemented with EDTA-free protease inhibitor cocktail (Complete, Roche, Mannheim, Germany)) by grinding (2x) with a combination of 2.3 mm zircon/silica and 3.2 mm stainless steel beads (BioSpec Products, Bartlesville, OK, USA) in a bead beater (1.5 min, 22.5 Hz; TissueLyser) with a 30 s interval between shakings. Samples were centrifuged for 10 min at 8000 rpm and the supernatant was transferred to a new tube. Protein concentration was measured using the BCA protein assay (Thermo Fisher Scientific) and samples were stored at −20 °C until further analysis.

### EnzChek gelatinase/collagenase assay

The Molecular Probes EnzChek® Gelatinase/Collagenase Assay Kit was used to evaluate the breakdown of gelatin, collagen type I and collagen type IV by enzymes present in the small intestinal tissues (duodenum, jejunum or ileum). These substrates were labeled with fluorescein and a quenching agent. Duplicate measurements were performed in 200 μL reaction volume containing 20 µL of either fluorescein labelled substrate (DQ Collagen I (25 μg/mL, D12060), DQ Collagen IV (25 μg/mL, D12052), or DQ Gelatin (12.5 μg/mL, D12054)), 100 μL of the tissue lysate (500 µg/mL) and 80 μL of reaction buffer (0.5 M Tris–HCl, 1.5 M NaCl, 50 mM CaCl_2_ and 2 mM sodium azide with pH 7.6). Samples were incubated for 10 h at room temperature in the absence of light, after which fluorescence was measured (excitation 485 nm, emission 527 nm; Fluoroskan Ascent Fluorometer, Thermo Fisher Scientific Inc.). Background fluorescence was subtracted for each sample.

### Gelatin substrate zymography

Gelatin zymography was used to identify the gelatinolytic enzymes in the ileal tissue lysates. Polyacrylamide gel (10%) containing 0.2% gelatin (2 mg/mL) as substrate was used for determination of MMPs gelatinolytic activity. Equal concentrations of ileal tissue lysates from the resin acids-supplemented birds or control birds were pooled, after which 10 µL pooled ileal tissue lysate (1 mg/mL) was mixed with 10 µL 2 × loading buffer (0.5 M Tris–HCl pH 6.8, 20% glycerol, 4% SDS, a pinch of bromophenol blue) and loaded to the gel. After standard electrophoresis, the gel was incubated with renaturing buffer (2.5% Triton X-100, 30 min, room temperature) to remove SDS from the gel. This allows the enzymes in the gels to renature and autoactivate. The gel was washed with developing buffer (150 mM NaCl, 5 mM CaCl_2_, 0.05% NaN_3_ and 50 mM Tris–HCl buffer pH 7.5) and incubated with fresh developing buffer under continuous shaking at 37 °C for 18 h. After incubation, the gel was stained with Coomassie brilliant blue (Sigma-Aldrich). Activity of gelatin-degrading enzymes is visualized as colorless bands on a blue background. Gel images were analyzed with a GS-800 calibrated densitometer and the Quantity One software (BioRad, Hercules, CA, USA).

### Statistical analysis

Statistical analysis on the gut microbiota were performed using R, as described above. All other analyses and calculations were performed using GraphPad Prism software (version 5.03, San Diego, CA, USA). Normality of the data was tested with the D’Agostino-Pearson normality test. A student’s *t*-test was used for normal distributed data. When the data were not normally distributed, the comparisons between groups were done by Mann–Whitney U test. Analyses were performed with 95% confidence intervals and significance was determined as *p* ≤ 0.05.

## Results

### Influence of resin acids on the caecal and ileal microbial diversity

**Figure 1:**
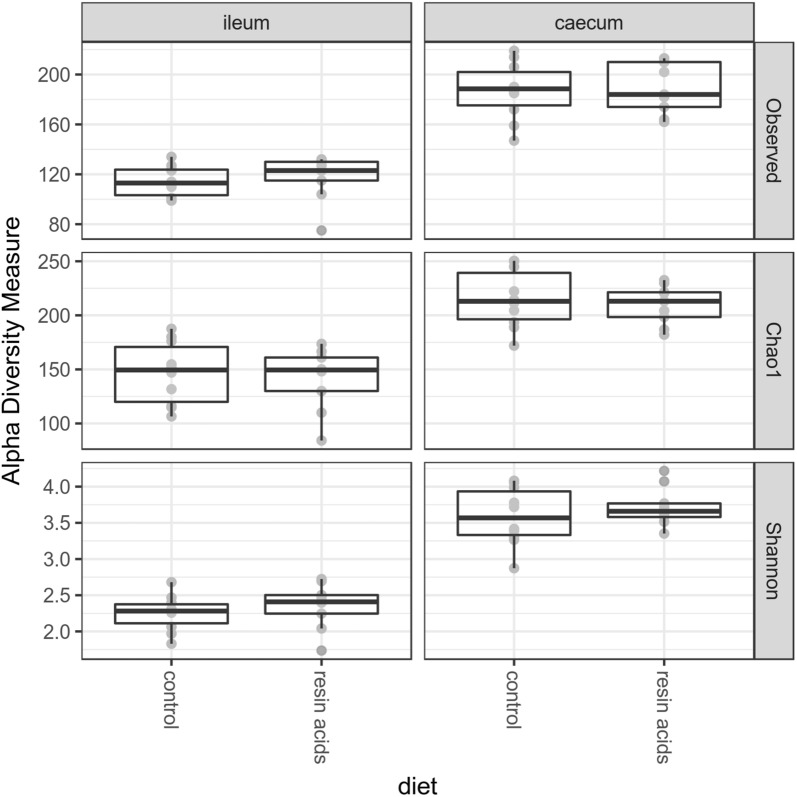
**Alpha diversity of the microbial community from birds fed a control or a resin acids-supplemented diet.** Observed: observed OTUs, Chao1: estimated OTU richness and Shannon: estimated community diversity.

The microbial complexity in the ileum and caecum of broiler chickens was estimated by calculating the number of observed OTUs, the estimated OTU richness (Chao1) or the estimated community diversity (Shannon index) in each sample. No significant alterations in either the caecal or ileal bacterial richness or diversity were introduced by supplementation of the diet with resin acids (Figure 1).

**Figure 2:**
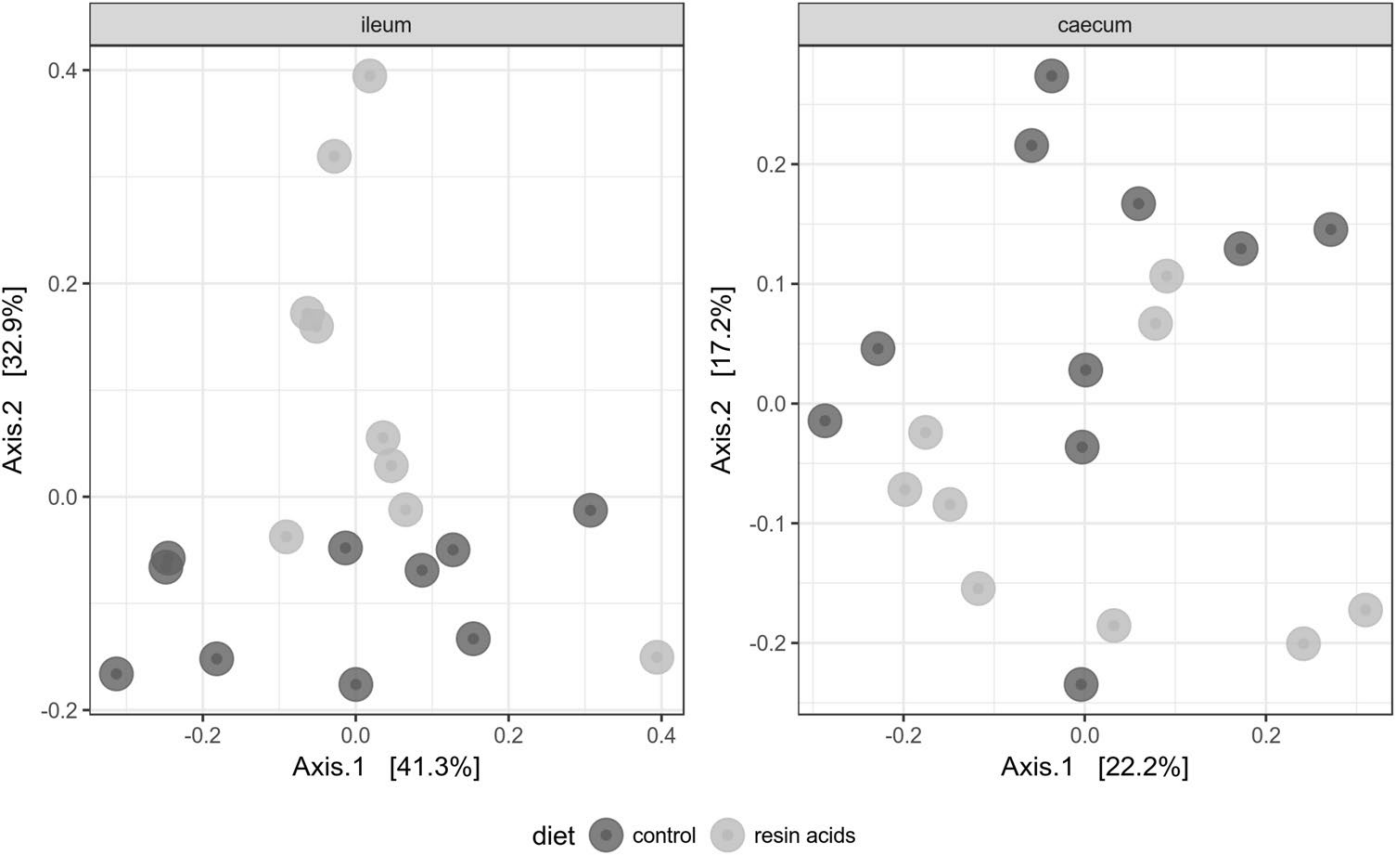
**PCoA plot of the microbiota from birds fed a control or resin acids-supplemented diet.** Principle coordinate analysis (PCoA) plot of Bray–Curtis dissimilarities. Each point represents a single chicken microbiome. Significant separation of ileal microbial communities were revealed using ANOSIM (*p* = 0.002). This difference was less pronounced in the caecal microbiota (*p* = 0.055).

Bray–Curtis dissimilarity was used to investigate beta diversity between either the caecal or ileal microbiota from birds fed the control diet or the diet supplemented with resin acids. Addition of resin acids to the diet resulted in a significant differentiation of the ileal microbial community structures as compared to the control group (ANOSIM statistic R = 0.23, *p* = 0.002), whereas no statistical difference could be observed in the caecum (ANOSIM statistic R = 0.12, *p* = 0.055) (Figure 2).

### Influence of resin acids on the taxonomic composition of the ileal and caecal microbiota

**Figure 3:**
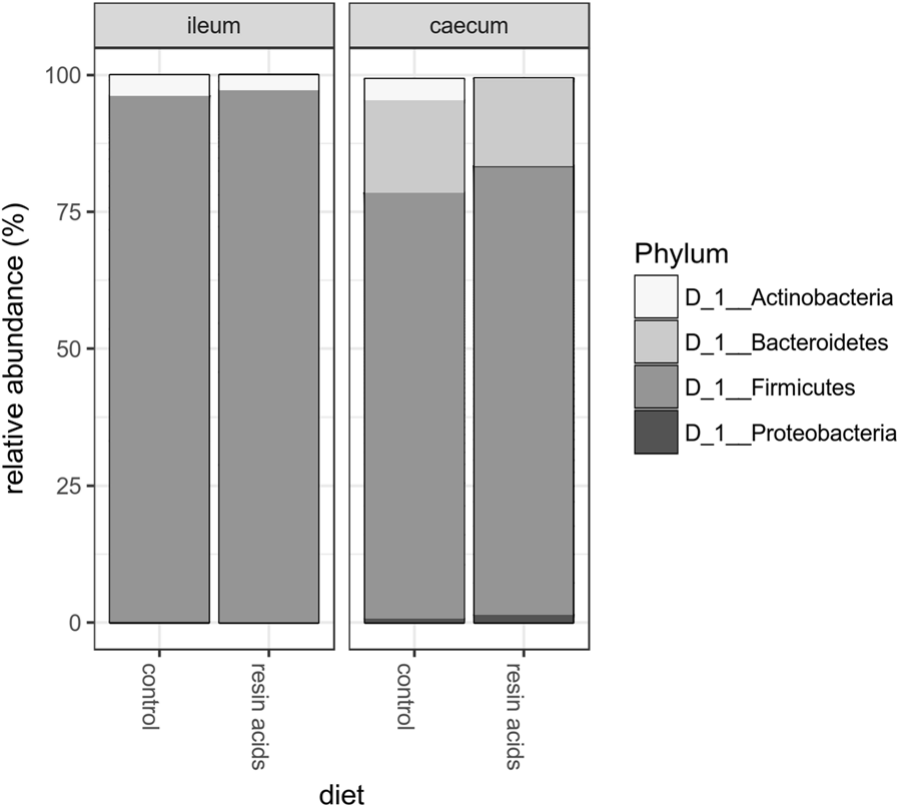
**Relative abundance at phylum level in the ileum or caecum.** Relative abundance (%) of the 4 most abundant phyla in the ileum or caecum from birds fed either the control diet or resin acids-supplemented diet.

The caeca from broilers fed a resin acids-containing diet or the control diet were highly abundant in *Firmicutes* (83.2% and 77.2%, respectively) and *Bacteroidetes* (14.5% and 17.8%, respectively). The relative abundance of *Actinobacteria* in the caeca from birds fed a resin acids-supplemented diet (0.07%) was decreased as compared to birds receiving the control diet (3.4%) (*p* = 0.0042), a difference that was mainly due to the genus *Bifidobacterium* (0.004% and 3.4% in respectively the resin acids group or control group). The phylum *Proteobacteria* accounted for 0.7% of total sequences in the control birds, whereas a relative abundance of 1.8% was observed in the caeca from birds receiving resin acids (*p* = 0.1992). The ileal microbiota was characterized by a high abundance of *Firmicutes* (97% in resin acids supplemented group, 96.1% in controls), followed by *Actinobacteria* as the second most abundant group (3.7% in control, 2.8% in resin acid fed birds) (Figure 3).

Differentially abundant genera in the caecal or ileal microbiota from birds fed a resin acids-supplemented diet as compared to the control diet were identified using DESeq2. Seven genera were differentially abundant between the caecal microbiota derived from birds fed either the control or resin acids diet. Six of these genera were more prevalent when resin acids were supplemented to the diet, with two belonging to the family *Lachnospiraceae*, three classified as *Ruminococcaceae* and the sixth belonging to the genus *Lactobacillus.* Only the genus *Bifidobacterium* was decreased when birds were fed a resin acids-containing diet (Table 3).

**Table 3:** **Differentially abundant genera in the caecal or ileal microbiota**

Phylum	Class	Order	Family	Genus	Mean relative abundance		Log_2_ fold change	Adjusted *p*-value
					Control	Resin acids		
Ileum								
*Actinobacteria*	*Actinobacteria*	*Bifidobacteriales*	*Bifidobacteriaceae*	*Bifidobacterium*	0.028%	0.000%	−5.96	7.26E−14
*Actinobacteria*	*Actinobacteria*	*Micrococcales*	*Brevibacteriaceae*	*Brevibacterium*	0.067%	0.011%	−3.29	8.14E−06
*Actinobacteria*	*Actinobacteria*	*Corynebacteriales*	*Corynebacteriaceae*	*Corynebacterium 1*	2.898%	1.797%	−1.55	2.81E−02
*Firmicutes*	*Bacilli*	*Bacillales*	*Planococcaceae*	*Kurthia*	0.167%	0.000%	−8.81	5.59E−28
*Firmicutes*	*Bacilli*	*Lactobacillales*	*Carnobacteriaceae*	*Trichococcus*	0.114%	0.013%	−3.46	1.36E−07
*Firmicutes*	*Bacilli*	*Lactobacillales*	*Carnobacteriaceae*	*Jeotgalibaca*	0.048%	0.062%	−1.86	8.30E−03
*Firmicutes*	*Bacilli*	*Lactobacillales*	*Lactobacillaceae*	*Pediococcus*	0.006%	0.027%	1.84	2.40E−02
*Firmicutes*	*Clostridia*	*Clostridiales*	*Peptostreptococcaceae*	*Ambiguous_taxa*	0.095%	0.001%	−5.58	2.07E−04
*Firmicutes*	*Clostridia*	*Clostridiales*	*Clostridiaceae 1*	*Candidatus Arthromitus*	0.734%	0.318%	−1.99	1.62E−02
*Firmicutes*	*Clostridia*	*Clostridiales*	*Peptostreptococcaceae*	*uncultured*	0.003%	0.027%	2.99	7.91E−05
*Firmicutes*	*Erysipelotrichia*	*Erysipelotrichales*	*Erysipelotrichaceae*	*Turicibacter*	0.014%	0.002%	−3.12	4.92E−05
*Firmicutes*	*Erysipelotrichia*	*Erysipelotrichales*	*Erysipelotrichaceae*	*Erysipelatoclostridium*	0.005%	0.020%	1.65	1.50E−02
Caecum								
*Actinobacteria*	*Actinobacteria*	*Bifidobacteriales*	*Bifidobacteriaceae*	*Bifidobacterium*	3.369%	0.004%	−8.90	2.62E−27
*Firmicutes*	*Bacilli*	*Lactobacillales*	*Lactobacillaceae*	*Lactobacillus*	2.484%	4.904%	1.51	3.75E−02
*Firmicutes*	*Clostridia*	*Clostridiales*	*Ruminococcaceae*	*uncultured*	2.935%	5.833%	1.42	1.63E−02
*Firmicutes*	*Clostridia*	*Clostridiales*	*Ruminococcaceae*	*DTU089*	0.727%	1.257%	1.93	1.63E−02
*Firmicutes*	*Clostridia*	*Clostridiales*	*Lachnospiraceae*	*Tyzzerella*	0.129%	0.608%	2.76	2.63E−02
*Firmicutes*	*Clostridia*	*Clostridiales*	*Lachnospiraceae*	*Lachnoclostridium 5*	0.011%	0.083%	3.64	1.63E−02
*Firmicutes*	*Clostridia*	*Clostridiales*	*Ruminococcaceae*	*Ruminococcaceae UCG*−*004*	0.000%	0.040%	7.21	1.67E−03

Significant differences in genus level abundance in the caecal or ileal microbiota from birds fed the resin acid-supplemented diet as compared to the control diet. The taxonomic classification, the mean relative abundance and the log_2_ fold change (resin acids/control) of the DESeq2 normalized abundance of each genus are shown.

Addition of resin acids to the broiler diet resulted in 12 genera in the ileal microbiota that were differentially abundant as compared to the control diet. Nine of these genera were less abundant when birds were fed the resin acids-containing diet. Five of these genera showed a mean relative abundance below 0.1%. The four more abundant genera belonged to the families *Corynebacteriaceaea, Planococcaceae, Carnobacteriaceae* and *Clostridiaceae 1*. The three genera that were more prevalent in the resin acids-supplemented group all showed an overall low prevalence in the ileal microbiota (mean relative abundance < 0.1%) (Table 3). The genus *Bifidobacterium* was the only genus that was significantly different between the diet groups in both the ileal and caecal microbiota.

### Resin acids-supplementation of broiler feed had no effect on the microbial activity

To determine whether the resin acids-induced alterations of the microbiota might have an effect on the microbial functions, microbial pathways present in the ileal and caecal microbiome were in silico predicted. The main functional pathways predicted in both the ileal and caecal microbiome were involved in membrane transport, DNA replication and repair, amino acid metabolism and carbohydrate metabolism. No significant differences could be observed between the microbiota from birds fed a resin acid-supplemented diet as compared to the control diet (Additional file 1).

In addition to the predicted metabolic function, the SCFA concentration in the caecum was determined. Acetate was the major SCFA, followed by butyrate as the second most abundant metabolite in the caecal content. No statistical differences in SCFA content were found between the resin acid supplemented and control group (Table 4).

**Table 4:** **Effect of dietary resin acids-supplementation on SCFA concentration in the caecum at day 22**

	Diet	
	Control	Resin acids
Acetate	39.27 ± 3.52	39.58 ± 3.28
Propionate	3.66 ± 1.15	3.89 ± 1.56
Butyrate	22.77 ± 5.88	21.71 ± 5.81
Valerate	1.51 ± 0.12	1.48 ± 0.46

Data are presented as mean SCFA concentration (mM) ± SD.

### Resin acids did not affect intestinal morphology but reduced duodenal T-cell abundance

Villus height, crypt depth and villus to crypt ratios were measured at the level of the duodenum, as a read-out for the evaluation of intestinal health. Feeding of broilers with resin acids-containing diet had no effect on any of these parameters, which approximate the reference values for broilers (villus to crypt ratio of 8 for broilers at 23 days of age; [56]) (Table 5).

**Table 5:** **Effect of resin acids-supplementation on intestinal morphology and T-cell abundance of chickens on day 22**

	Diet		
	Control	Resin acids	*p*-value
Intestinal morphology (duodenum)			
Villus height (µm)	1708 ± 163	1738 ± 128	0.84
Crypt depth (µm)	229 ± 36	222 ± 23	0.90
Villus to crypt ratio	7.84 ± 1.55	8.13 ± 1.05	0.97
CD3 area percentage			
Duodenum	6.78 ± 2.12	4.78 ± 1.62	0.036
Ileum	14.54 ± 4.05	12.24 ± 4.88	0.27

Data represent the mean ± standard deviation. Analysis based on 10 measurements per section for villus height and crypt depth analysis or 3 microscopic fields of view per section for CD3 measurements.

The amount of CD3^+^ T-cells was determined in both the ileal and duodenal tissue as a marker for intestinal inflammation. No changes in ileal T-cell abundance were observed, whereas the amount of CD3^+^ positive cells in the duodenal tissue from birds fed the resin acids-supplemented diet was significantly decreased as compared to the control birds (Table 5).

### Resin acids-supplementation decreased the collagenolytic activity in the broiler ileal tissue

**Figure 4:**
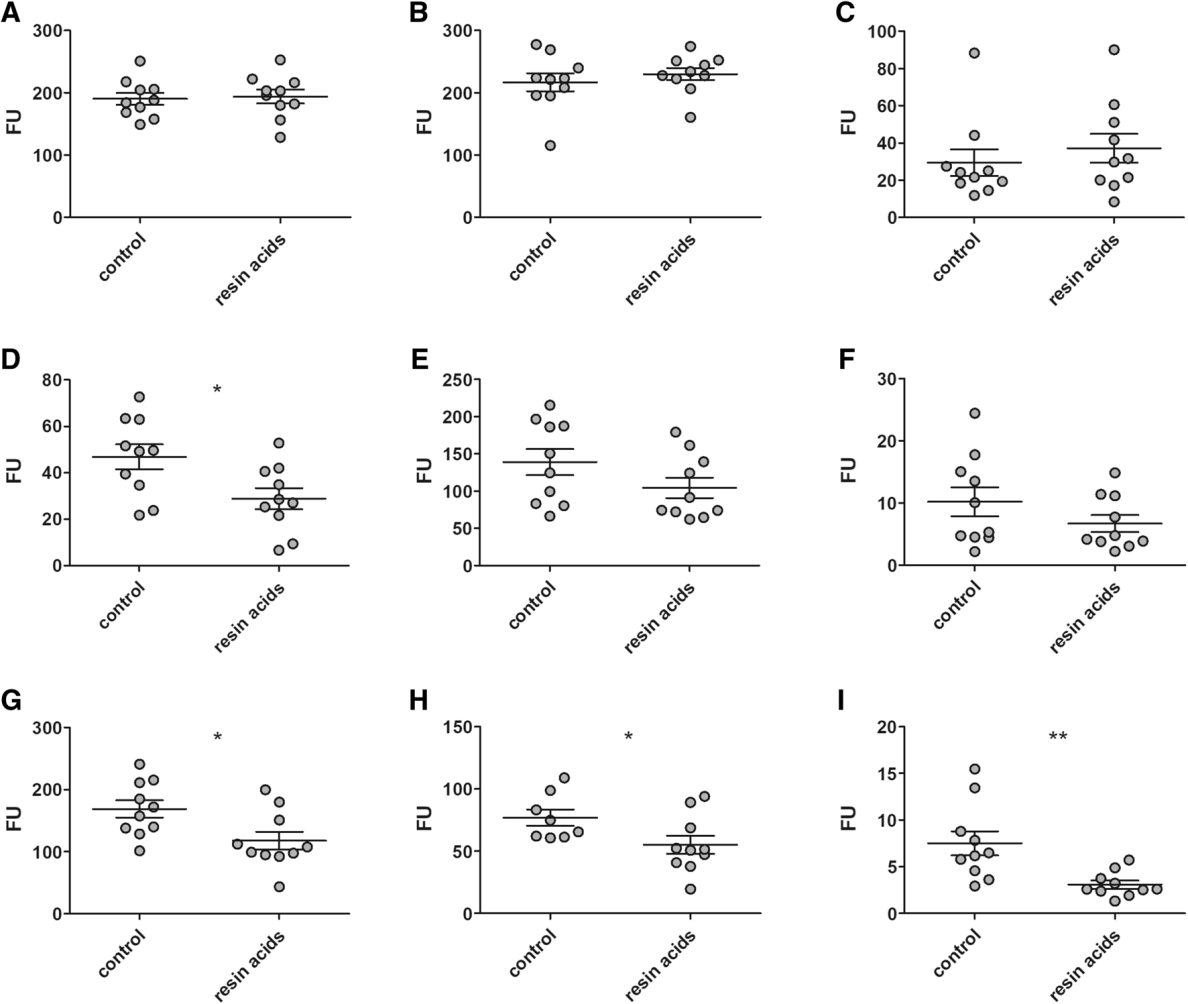
**Relative gelatin, collagen type I and collagen type IV degrading activity in small intestinal tissue.** Duodenal (**A**–**C**), jejunal (**D**–**F**) or ileal (**G**–**I**) intestinal tissue lysates from either control birds or resin acids fed birds were incubated for 10 h with fluorescently labelled gelatin (**A**, **D** and **G**), collagen type I (**B**, **E** and **h**) or collagen type IV (**C**, **F** and **I**). Breakdown of the fluorescently labelled substrate results in an increase of fluorescence which is proportional to the substrate degrading activity of the sample. Data represent the mean (± standard deviation). FU: relative fluorescence units after 10 h incubation. *p < 0.05, **p < 0.01.

The enzymatic activity towards gelatin, collagen type I or collagen type IV present in the small intestinal tissue was assessed as a measure for extracellular matrix degradation in the gut. Resin acids-supplementation did not influence the gelatin, collagen type I or collagen type IV degrading activity in the duodenal tissue (Figures 4A–C). In the jejunum, significantly lower gelatinase activity was measured in the tissue from resin acids fed birds as compared to the control group (*p* = 0.0197; Figure 4D). Although still numerically lower in the resin acids-supplemented group, this difference did not reach statistical significance when looking to the specific collagen type I or collagen type IV breakdown in the jejunal tissue (*p* = 0.14 or *p* = 0.21 respectively, Figures 4E and F). The biggest effect of resin acids was observed in the ileal tissue, where gelatin (*p* = 0.021), collagen type I (*p* = 0.046) and collagen type IV (*p* = 0.0045) degrading activity was significantly decreased by supplementation of resin acids to the broiler feed (Figures 4G–I).

**Figure 5:**
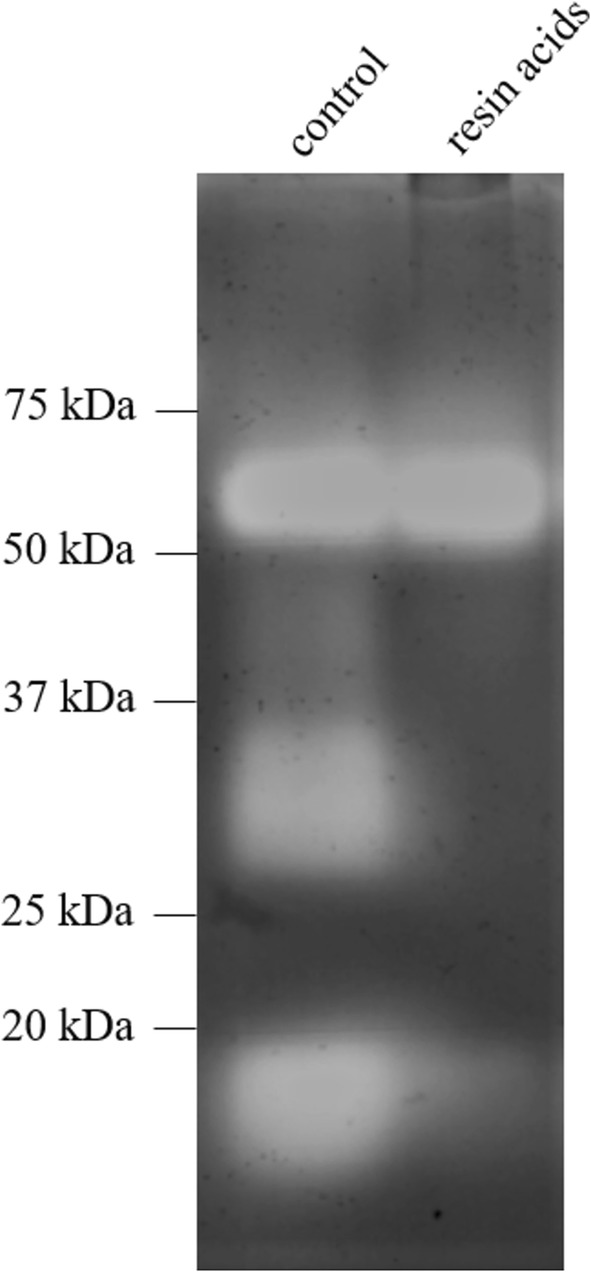
**Effect of resin acids-supplementation on ileal gelatinolytic enzymes.** Ileal tissue lysates from either the control group or the resin acids-supplemented group were pooled and subjected to gelatin zymography. Gelatinolytic enzymes are observed as clear bands of digested gelatin. Control tissue shows three different gelatinolytic bands, whereas only one band was observed in the ileal tissue lysate from birds fed the resin acids-containing diet.

To gain more information on the identity of these enzymes, pooled ileal tissue lysates were subjected to gelatin zymography (Figure 5). The ileal tissue from the control birds showed three different gelatinolytic bands, whereas in the ileal tissue from the resin acids group, only the highest molecular weight enzyme was present. The two enzymatic bands that were exclusively detected in the ileum from control birds correspond to MMP7 (~18 kDa) and its latent pro-enzyme forms (pre-proMMP7: ~30 kDa and proMMP7: ~28 kDa) (UniprotKB: F6R1W4_CHICK; [57]). MMP7 is the smallest of all MMPs and the only MMP in this molecular weight range. The third, unaffected enzymatic band has a molecular weight between 50 and 75 kDa, which is predicted to be MMP2 (~62 kDa), but might as well be MMP1 (54 kDa), MMP3 (52 kDa) or MMP13 (55 kDa). As this bigger enzyme was not affected by resin acids-supplementation, the identity of this enzyme was not further investigated.

## Discussion

Feed additives are widely used to improve broiler gut health and to stimulate performance. Dietary inclusion of a resin-based product containing a combination of tall oil fatty acids (~90%) and resin acids (8–9%) from coniferous trees has recently been shown to improve broiler performance, but the mechanism behind this effect is still unknown [35–37]. In the present study, we demonstrated that administration of pure resin acids to broiler feed has a major effect on the host intestinal tissue, with only minor effects on the microbiota composition in the gut.

Despite the known anti-microbial properties of resin acids extracted from coniferous trees [20, 23, 58], dietary inclusion of resin acids had no effect on the microbial richness or diversity in either the ileum or caecum, nor did it alter the overall microbial community composition of the caecum. The composition of the ileal microbial structure did show differences between the resin acids-supplemented group and control birds, without affecting the composition of the caecal microbial structure. This is consistent with previous studies using either plant derived growth promoters or antimicrobial growth promoters, where the major microbial responses were observed in small intestinal compartments rather than in caecal or colorectal compartments [59]. In the current study, the overall effects on ileal microbial composition were mainly due to low abundant genera (< 0.1% relative abundance). Moreover, no effect on the metabolic microbial functions were detected (both as inferred functional capacities of the microbial communities as well as measured SCFA production), indicating that dietary supplementation of resin acids had likely no relevant effect on the microbiota of healthy broilers.

Intestinal homeostasis is the result of a delicate balance between toxic and inflammatory elements in the gut that constantly challenge its integrity on the one hand (e.g. antigens, toxins, invasive bacteria), and beneficial microbes and host signals that are supporting intestinal integrity and regulating immunity on the other hand [2, 38, 60]. In the ideal situation, the intestinal wall is covered by a protective layer of mucins and the intestinal epithelial cells are strongly sealed by intercellular junctions. Additionally, there exists a symbiotic homeostasis between the intestinal microbiome and the intestinal immune system, inducing a low-grade stimulation of the innate immune system that is continuously regulated and controlled, thereby preventing intestinal tissue damage [60]. To ensure optimal performance, normal physiological inflammation should be maintained while limiting pathological inflammatory triggers. However, features of modern animal production likely predispose broilers to particularly chronic, inflammatory triggers (e.g. increased feed intake and nutrient excesses) [60]. Anti-inflammatory nutritional strategies and other strategies that support optimal gut barrier function are important means to improve the performance of broilers. By reducing the inflammatory tone, the production of protein can be assigned to support growth instead of to the production of immune modulators [61]. The anti-inflammatory and wound healing activities described for coniferous resin acids might be beneficial to maintain broiler gut health and can be a possible explanation for the previously reported performance-enhancing effects. Indeed, dietary resin acids-supplementation resulted in decreased abundance of inflammatory T-cells in the duodenal tissue and reduced matrix metalloproteinase (MMP) activity, while maintaining optimal intestinal morphology. MMPs are zinc-dependent endopeptidases that are able to degrade extracellular matrix molecules as well as a wide range of other molecules that might be important within the mucosal layer, such as, amongst others, membrane receptors, adhesion factors, signaling molecules and cytoskeleton proteins [62]. MMPs are important in many normal physiological processes, but some MMPs are also involved in various enteric inflammatory diseases, such as inflammatory bowel disease [62, 63] and necrotic enteritis in broilers [64]. The most profound effect of resin acids-supplementation on MMP activity was observed in the ileum, resulting in a reduction of both collagen type I and collagen type IV degrading activity. Both collagen subtypes are important for the structural integrity of the intestinal wall: collagen type I is a major supportive component of the extracellular matrix, whereas type IV collagen is an integral component of the basement membrane supporting the epithelial cells [65, 66]. The main enzyme responsible for the reduced collagenolytic activity in the resin acids group had a molecular weight corresponding to both the latent and active forms of MMP7. No other MMPs in this molecular weight range are currently described. However the identity of this enzyme should be confirmed in future research. In the healthy intestine, various MMPs are expressed, but the production of MMP7 is mainly linked to injured epithelium and seems not to be involved in regular epithelial renewal [67, 68]. MMP7 can disrupt epithelial barrier integrity by degrading intercellular junction molecules such as cadherins and occludins. Furthermore, MMP7 is able to activate α-defensins by cleaving its precursor into the active form. Αlpha-defensins are antimicrobial peptides that are secreted by epithelial cells and granulocytes, which might play a role in protection of the host against microbial invasion during intestinal inflammation [69] and can induce IL6 secretion by macrophages [70]. While IL6 has a protective role in many infections, these effects are context dependent, and the same activities can also contribute to intestinal leakage and inflammation. As the broiler gut is continuously exposed to various challenges that affect intestinal barrier integrity and can trigger inflammation (e.g. coccidia, mycotoxins, bacterial toxins, amongst others), reducing MMP7 activity through resin acids-supplementation to the diet might support intestinal health by enhancing intestinal barrier integrity and controlling inflammation in the avian gut.

In the present study, resin acids-supplementation of broiler diet reduced small intestinal MMP activity and duodenal inflammatory T-cell abundance, while maintaining optimal gut morphology. Only minor effects on the microbiota composition and its metabolic functions were observed. To confirm the presumably direct effects of resin acids on the propria mucosa homeostasis, further in vitro studies are required in absence of the host microbiota. The observation that resin acids have an effect on host intestinal inflammation and MMP activity provides a new direction for future research on the effects of resin acids on broiler intestinal health.

## Additional file

**Additional file 1.** **In silico predicted KEGG pathway abundances in the ileal and caecal microbiota at day 22.**

## Abbreviations

MMP: matrix metalloproteinase
OTU: operational taxonomic units
PCoA: principle coordinate analysis
SCFA: short-chain fatty acids
SD: standard deviation
