# Supplementary material for: In-feed resin acids reduce matrix metalloproteinase activity in the ileal mucosa of healthy broilers without inducing major effects on the gut microbiota
Source: Vet Res. 2019 Feb 22;50:15. doi: 10.1186/s13567-019-0633-3 (PMC6387527; doi:10.1186/s13567-019-0633-3)
Supplement: Supplementary file 1 — Additional file 1. In silico predicted KEGG pathway abundances in the ileal and caecal microbiota at day 22. [file 13567_2019_633_MOESM1_ESM.docx]

| **KEGG pathway** | **Ileum** | | **Caecum** | |
| --- | --- | --- | --- | --- |
|  | **control** | **resin acids** | **control** | **resin acids** |
| **Cellular Processes** | | | | |
| Cell Growth and Death | 0.608% | 0.598% | 0.555% | 0.538% |
| Cell Motility | 0.641% | 0.654% | 1.404% | 1.581% |
| Transport and Catabolism | 0.108% | 0.116% | 0.219% | 0.198% |
| **Environmental Information Processing** | | | | |
| Membrane Transport | 13.991% | 13.569% | 12.405% | 12.417% |
| Signal Transduction | 1.250% | 1.384% | 1.267% | 1.317% |
| Signaling Molecules and Interaction | 0.264% | 0.254% | 0.137% | 0.132% |
| **Genetic Information Processing** | | | | |
| Folding, Sorting and Degradation | 2.384% | 2.343% | 2.445% | 2.393% |
| Replication and Repair | 11.040% | 10.854% | 9.365% | 9.307% |
| Transcription | 2.691% | 2.745% | 3.121% | 3.172% |
| Translation | 7.172% | 7.072% | 5.941% | 5.854% |
| **Human Diseases** | | | | |
| Cancers | 0.110% | 0.106% | 0.101% | 0.102% |
| Immune System Diseases | 0.088% | 0.087% | 0.051% | 0.050% |
| Infectious Diseases | 0.475% | 0.469% | 0.341% | 0.338% |
| Metabolic Diseases | 0.117% | 0.115% | 0.112% | 0.111% |
| Neurodegenerative Diseases | 0.077% | 0.075% | 0.077% | 0.071% |
| **Metabolism** | | | | |
| Amino Acid Metabolism | 6.976% | 7.129% | 9.952% | 9.954% |
| Biosynthesis of Other Secondary Metabolites | 0.567% | 0.517% | 0.923% | 0.891% |
| Carbohydrate Metabolism | 11.469% | 11.464% | 11.330% | 11.417% |
| Energy Metabolism | 5.035% | 5.061% | 5.634% | 5.540% |
| Enzyme Families | 2.304% | 2.264% | 2.210% | 2.198% |
| Glycan Biosynthesis and Metabolism | 1.899% | 1.880% | 1.772% | 1.747% |
| Lipid Metabolism | 2.893% | 2.957% | 2.999% | 3.041% |
| Metabolism of Cofactors and Vitamins | 3.155% | 3.442% | 4.279% | 4.209% |
| Metabolism of Other Amino Acids | 1.568% | 1.604% | 1.303% | 1.282% |
| Metabolism of Terpenoids and Polyketides | 1.787% | 1.802% | 1.613% | 1.605% |
| Nucleotide Metabolism | 5.161% | 5.094% | 4.090% | 4.036% |
| Xenobiotics Biodegradation and Metabolism | 2.459% | 2.548% | 1.732% | 1.811% |
| **Organismal Systems** | | | | |
| Digestive System | 0.016% | 0.015% | 0.025% | 0.027% |
| Endocrine System | 0.127% | 0.131% | 0.334% | 0.332% |
| Environmental Adaptation | 0.131% | 0.140% | 0.159% | 0.166% |
| Excretory System | 0.026% | 0.030% | 0.017% | 0.020% |
| Immune System | 0.012% | 0.013% | 0.096% | 0.095% |
| Nervous System | 0.087% | 0.090% | 0.105% | 0.105% |
| **Unclassified** | | | | |
| Cellular Processes and Signaling | 3.149% | 3.225% | 4.171% | 4.208% |
| Genetic Information Processing | 2.819% | 2.847% | 2.711% | 2.700% |
| Metabolism | 2.308% | 2.308% | 2.386% | 2.393% |
| Poorly Characterized | 5.037% | 4.999% | 4.619% | 4.641% |

Control: microbiota of birds fed a control diet, resin acids: microbiota of birds fed a resin acids-containing diet
